# Integrated metabolomics, network pharmacology and molecular docking to reveal the effects of drying processes on the antioxidant activity of mulberry (*Morus alba* L.) leaves

**DOI:** 10.3389/fpls.2026.1843809

**Published:** 2026-06-08

**Authors:** Yang Liu, Wendan Chi, Qian Liu

**Affiliations:** 1 Haide College, Ocean University of China, Qingdao, Shandong, China; 2 Marine Sciences Research Institute of Shandong Province, Qingdao, Shandong, China

**Keywords:** antioxidant, drying method, integrated metabolomics, molecular docking, mulberry leaves, network pharmacology

## Abstract

**Introduction:**

Mulberry (*Morus alba* L.) leaves (MLs), a well-known food-medicine homologous substance, possess potent properties against oxidative stress. However, the impact of industrial drying methods on their antioxidant activity is still not completely clear.

**Methods:**

A comprehensive approach based on UPLC-MS/MS metabolomics, network pharmacology and molecular docking was employed to find the active compounds in MLs treated by different drying methods and their potential antioxidant mechanisms in this study.

**Results and discussion:**

A total of 1467 metabolites were detected through UPLC-MS/MS analysis, and differential metabolites of MLs were searched by multivariate statistical analysis. Hot-air dried MLs exhibited the highest DPPH and ABTS radical scavenging capacities, as well as the strongest ferric reducing antioxidant power. This enhanced activity might be attributed to the maximal hydrolysis of kaempferol glycosides, leading to the significant accumulation of free kaempferol. Network pharmacology analysis further revealed that the bioactive compounds in MLs exerted their antioxidant effects by targeting key proteins, including AKT1, TNF, ALB, IL-1B, and BCL2. These findings provided valuable insights into how different drying methods influenced the antioxidant property of MLs.

## 1 Introduction

Reactive oxygen species (ROS) is a collective term for oxygen-derived free radicals, including hydroxyl radicals, singlet oxygen, and superoxide anions. Mitochondria, the principal venue for ROS production within the organism, generate ROS primarily via energy metabolism (Zorov et al., 2014). In modern society, the human body faces challenges from multiple external environmental factors, such as environmental pollution, intense ultraviolet radiation, and exposure to toxic substances, which significantly increase the production of endogenous ROS. Furthermore, improper dietary habits, such as nutritional imbalance and high-sugar, high-fat diets, also promote elevated ROS levels. While low levels of ROS are beneficial for physiological functions like cell signaling, immune defense, and gene expression regulation, excessive ROS levels exceed the body’s antioxidant defense capacity, causing oxidative stress, cellular damage, and even cell death (Mittal et al., 2014). Under conditions of oxidative stress, excessive ROS damages biological macromolecules, leading to the peroxidation of proteins and lipids, as well as structural DNA damage (Schieber and Chandel, 2014). If excessive ROS are not scavenged or their production is not inhibited promptly, the body remains in a persistent state of oxidative stress, which is considered a significant inducer of aging and various chronic diseases. Previous studies had demonstrated a close relationship between oxidative stress and the pathogenesis of numerous chronic conditions, such as cardiovascular diseases, neurodegenerative diseases, and diabetes (Ogihara et al., 2004; Choi et al., 2024). Therefore, inhibiting the excessive production of ROS and enhancing the body’s antioxidant capacity are important strategies for preventing and alleviating chronic diseases.

Recent research indicated that substances such as polyphenols (Zeng et al., 2025), flavonoids (Cao et al., 2023), and triterpenes (Lim et al., 2024) found in plants possessed excellent antioxidant activities and were potential bioactive agents for reducing oxidative stress.

Mulberry leaves (MLs), the leaves of the *Morus alba* L., are a typical substance that is traditionally both food and Chinese herbal medicines. They were widely consumed due to their potential efficacy in antioxidant activity (Chauhan et al., 2013), anti-inflammatory effects (Lin et al., 2022), and hypoglycemic properties (Zhang et al., 2024). *Morus alba* L. are widely cultivated across temperate and tropical regions. China is the world’s leading mulberry cultivator, accounting for over 75% of the global cultivation area. MLs had a complex chemical profile rich in diverse bioactive compounds, including flavonoids and their glycosides, alkaloids, phenylpropanoids, steroids, triterpenes, volatile oils, and amino acids (Chen et al., 2022). Typically, the MLs were generally harvested after frost. There were significant differences between frost-bitten and ordinary MLs in terms of chemical composition and biological activity, particularly as low temperatures promoted the accumulation of flavonoids (Yang et al., 2023).

Drying was necessary for fresh MLs because they had a high moisture content and were difficult to preserve. The nutritional components of MLs were significantly influenced by their drying methods. Previous study demonstrated that oven drying effectively retained amino acids, whereas freeze-drying and microwave drying preserved more vitamin C and flavonoids. Moreover, microwave drying had the best retention effect on minerals, and microwave-dried MLs exhibited a stronger ability to resist lipid accumulation and oxidative stress (Zhao et al., 2025). In addition, another research found that the polysaccharide yield of freeze-dried MLs was approximately 28.88% higher than that of hot-air dried MLs (Ma et al., 2018). Stir-frying, a traditional processing method, has been applied to various types of plant processing. However, the impact of stir-frying differed among various plants. It was reported that the contents of amino acids and total flavonoids in tartary buckwheat bran decreased after baking at 180 °C for 30 min (Ge and Wang, 2020). On the contrary, stir-frying changed the polyphenols composition and significantly improved the xanthine oxidase inhibitory activity of *Flos Sophorae Immaturus* tea (Li et al., 2023).

At present, stir-frying drying, hot-air drying and freeze-drying are still the most widely used methods in the plant industry. Although there were some studies investigated the effects of different drying methods on the nutritional components of MLs, no study had yet incorporated the stir-frying drying method for comparison.

Therefore, this study employed a comprehensive approach combining “intargeted metabolomics based on UPLC-MS/MS, network pharmacology, and molecular docking” to analyze the effects of three drying methods on the composition and antioxidant activity of MLs, and elucidated the underlying mechanisms. These results will enhance our understanding of the active compounds in MLs and their antioxidant mechanisms, facilitating the development of optimized drying techniques for MLs.

## 2 Materials and methods

### 2.1 Materials

The frost-bitten MLs were sourced from Rizhao, Shandong, China. 2,4,6-tripyridyl-s-triazine (TPTZ), 6-hydroxy-2,2-2,5,7,8-tetramethylchromane-2-carboxylic acid (Trolox), and 2,2-Azinobis-3-ethylbenzothiazoline 6-sulphonate (ABTS) were obtained from Beijing Solarbio Science & Technology Co., Ltd, 2,2-diphenyl-1-picrylhydrazyl (DPPH) was purchased from Tokyo Chemical Industry Co., Ltd.

### 2.2 Preparation of dried MLs

The harvested MLs were immediately dried using three different methods.

Freeze-drying (FD): the MLs were pre-frozen at −80 °C for 12 h and subsequently freeze dried in a lyophilizer (FDL-2000, EYELA, Tokyo, Japan) under vacuum pressure of 0.002 kPa for 48 h.

Hot-air drying (HD): the MLs were placed in a hot-air oven (DHG-9145A, Shanghai Yiheng Scientific Instrument Co., Ltd, Shanghai, China) at 65 °C for 8 h with an air velocity of 1.5 m/s.

Stir-frying drying (SD): MLs were processed in an electric roasting machine (6CCT-80, Zhejang Chunjiang Tea Machinery Co., Ltd, Hangzhou, China) preheated to 120 °C and stir-fried at 60 rpm for 20 min.

After drying, the moisture content of all samples was <8.0%. The dried MLs were ground into a fine powder in liquid nitrogen and stored at −80 °C until further analysis.

### 2.3 Preparation of active compounds from MLs

Dried ML powder (20 mg ± 1 mg) was combined with extraction beads and 1000 μL of a solvent mixture (MeOH/ACN/H_2_O, 2:2:1, v/v) containing deuterium-labeled internal standards. The mixture was vortexed for 30 s, homogenized at 35 Hz for 4 min, and sonicated in a 4 °C water bath for 5 min. This homogenization–sonication cycle was repeated three times. The samples were then incubated at −40 °C for 1 h to promote protein precipitation. Afterward, the extracts were centrifuged at 12,000 rpm for 15 min at 4 °C. A total of 400 μL of the resulting supernatant was transferred to a protein precipitation plate and processed under vacuum at 6 psi for 120 s. The filtrates were collected for subsequent untargeted metabolomics detection.

The ML extracts for antioxidant activity evaluation were prepared following the same protocol as above, except that no deuterium-labeled internal standards were added to the extraction solvent mixture (MeOH/ACN/H_2_O, 2:2:1, v/v).

### 2.4 Untargeted metabolomics analysis using LC-MS/MS

LC–MS/MS analysis of non-polar metabolites was performed using UHPLC system (Vanquish, Thermo Fisher Scientific, Massachusetts, America) equipped with a Phenomenex Kinetex C18 column (2.1 × 100 mm, 2.6 μm) and coupled to a mass spectrometer (Orbitrap Exploris 120, Thermo Fisher Scientific, Massachusetts, America). The mobile phase consisted of solvent A (0.01% acetic acid in water) and solvent B (IPA/ACN, 1:1, v/v). The column temperature was maintained at 25 °C, the autosampler was kept at 4 °C, and the injection volume was 2 μL. The Orbitrap Exploris 120 was operated in information-dependent acquisition mode using Xcalibur software to enable real-time selection of precursor ions for MS/MS fragmentation. Electrospray ionization parameters were set as follows: sheath gas 50 Arb, auxiliary gas 15 Arb, sweep gas 1 Arb, capillary temperature 320 °C, and vaporizer temperature 350 °C. Full MS and MS/MS spectra were acquired at resolutions of 60,000 and 15,000, respectively. Stepped normalized collision energies of 20, 30, and 40 were applied for fragmentation. Spray voltages were set to 3.8 kV in positive ion mode and −3.4 kV in negative ion mode. According to the Metabolomics Standards Initiative (MSI), the annotations were at Level 1, Level 2, and Level 3. Total ion current normalization method was employed in this data analysis. The final dataset containing the information of feature number, sample name and normalized feature area was imported to SIMCA18.0.1 software package (Sartorius Stedim Data Analytics AB, Umea, Sweden) for multivariate analysis.

### 2.5 Determination of *in vitro* antioxidant activity

#### 2.5.1 DPPH radical scavenging assay

The DPPH radical scavenging activity was evaluated using a modified spectrophotometric method. A 0.1 mM DPPH solution was freshly prepared in anhydrous ethanol. The sample solution was mixed with the DPPH solution at a 1:10 ratio (v/v), and incubated in the dark at room temperature for 30 min. The absorbance of the reaction mixture was then measured at 517 nm, using anhydrous ethanol as the blank.

#### 2.5.2 ABTS radical scavenging assay

The ABTS radical scavenging activity was determined through a modified decolorization assay. ABTS•^+^ stock solution was generated by reacting 7.0 mM ABTS solution with 2.45 mM potassium persulfate and incubating the mixture in the dark for 12–16 h. Before use, the ABTS•^+^ stock solution was diluted with distilled water to achieve an absorbance of 0.70 ± 0.02 at 734 nm. The sample solution was mixed with the ABTS•^+^ working solution at a 1:10 ratio (v/v) and the mixture was incubated at room temperature for 6 min. Absorbance was recorded at 734 nm using distilled water as the blank control.

#### 2.5.3 Ferric reducing antioxidant power assay

The FRAP reagent was freshly prepared by combining NaAc-HAc buffer (300 mM, pH 3.6), 10 mM of 2,4,6-Tripyridyl-s-triazine solution (in 40 mM HCl) and 20 mM of FeCl_3_·6H_2_O in a 10:1:1 (v/v) ratio. The sample solution was mixed with pre-warmed FRAP reagent at a 1:20 (v/v) ratio and reacted at 37 °C for 10 min. The absorbance at 593 nm was measured using a multimode microplate reader (Varioskan LUX, Thermo Fisher Scientific, Massachusetts, America).

Trolox was used as the positive control in above assays, and a corresponding calibration curve was constructed. The results were expressed as trolox equivalent per gram of sample (mg Trolox/g DW).

### 2.6 Network pharmacology analysis

#### 2.6.1 Screening of potential bioactive compounds

Potential antioxidant-active compounds were screened based on the structure of the compounds. The SMILE string of chosen ML metabolites was obtained from the PubChem database (https://pubchem.ncbi.nlm.nih.gov/).

#### 2.6.2 Target prediction for potential bioactive compounds and disease

The targets of potential active compounds were obtained from Swiss Target Prediction database (http://swisstargetprediction.ch/). To ensure refined selection, targets with a “probability ≥ 0.1” were included in the subsequent analysis. The keyword “oxidative stress” was used to retrieve disease-associated genes from databases including OMIM (https://www.omim.org/), GeneCards (https://www.genecards.org/) and TTD (https://ttd.idrblab.cn/).

#### 2.6.3 Construction of “MLs-compounds-targets” network and PPI network

A venn analysis was used to find compound-disease common targets. Then, the common targets were submitted to the STRING database (https://cn.string-db.org/) for protein–protein interaction (PPI) analysis. Subsequently, the core targets were selected based on degree, closeness, and betweenness values exceeding their respective averages using Cytoscape 3.10.0. The “MLs-compounds-targets network” was also generated in Cytoscape, with node and edge size and color representing degree values and combined scores.

#### 2.6.4 Gene ontology and kyoto encyclopedia of genes and genomes analysis

To elucidate the functions and metabolic pathways of core genes, the David database (https://david.ncifcrf.gov/) was used for GO (biological process, cellular component, molecular function) and KEGG pathway analysis. Then the visualization of GO and KEGG analysis results was conducted in bioinformatics website (https://bioinformatics.com.cn/).

### 2.7 Molecular docking

The 3D structure of GAPDH (PDB ID: 6YNE), AKT1 (PDB ID: 1H10), TNF (PDB ID: 1A8M), ALB (PDB ID: 9IK6), IL-1B (PDB ID: 5R87), BCL2 (PDB ID: 1G5M) was downloaded from the Protein Data Bank database (https://www.rcsb.org/), and the information of structure quality was shown in **Supplementary Table 1**. Water molecules, heteroatoms and redundant chains were removed using PyMOL, and the target chain was retained and hydrogenated. The SDF structure of the active compounds was obtained from PubChem, and subjected to energy minimization, hydrogenation and charge assignment in Discovery Studio. Molecular docking was performed using AutoDock Vina, with the docking pocket defined according to the active site of the protein. After docking, the binding energy was extracted from the log file, and the results were visualized using PyMOL.

### 2.8 Statistical analysis

All experiments were performed in triplicate, and the results were presented as mean ± standard deviation (SD). Statistical analysis was conducted by ANOVA and Tukey’s multiple comparison tests using GraphPad Prism 8 (GraphPad Software, San Diego, CA, USA). For untargeted metabolomics data processing, features exhibiting identical retention times and peak areas were considered unresolved co-eluting compounds. To prevent statistical bias and false positives, these features were merged into a single co-elution group prior to differential analysis. Principal component analysis (PCA), orthogonal partial least squares discriminant analysis (OPLS-DA), Pearson correlation analysis and data visualization of metabolomics data were performed using the Metware Cloud (https://cloud.metware.cn). Venny 2.1.0 was used to perform venn analysis.

## 3 Results

### 3.1 Metabolite profile of dried MLs

**Figure 1:**
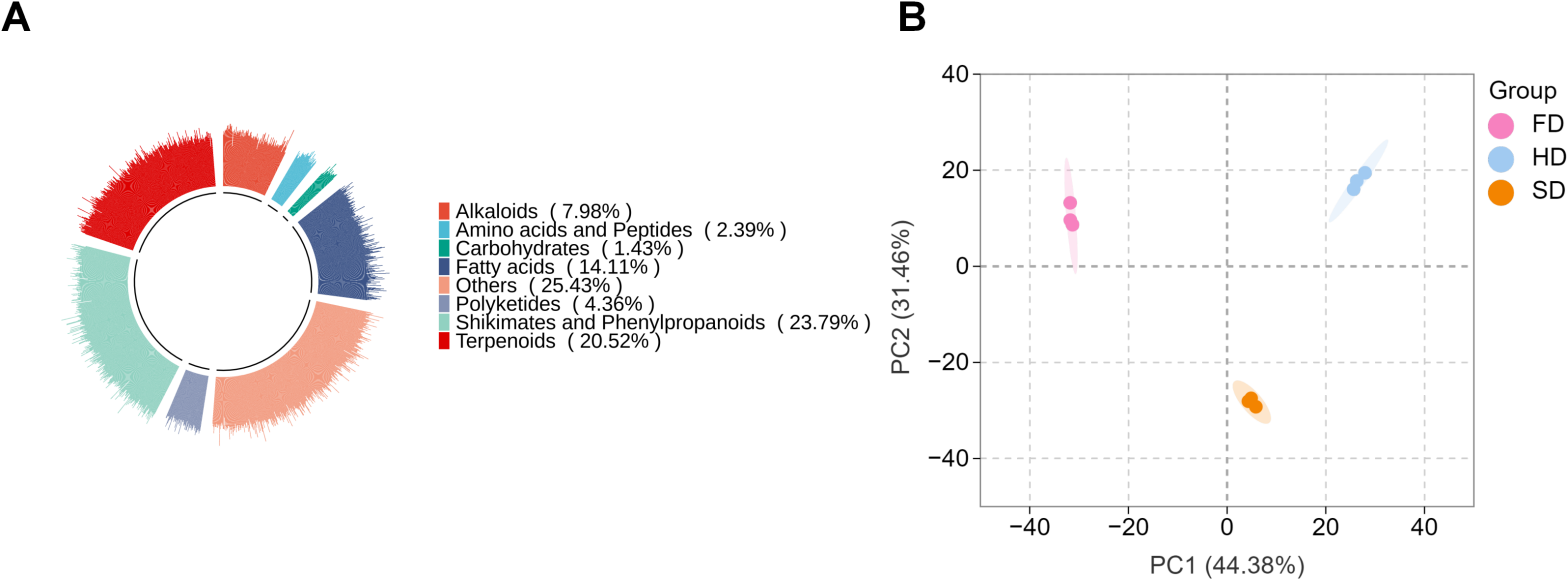
**(A)** Classification of the 1467 metabolites of MLs. **(B)** PCA score plot. The sampling groups were color-coded as follows: red, FD; blue, HD; yellow, SD.

The metabolomics data was shown in **Supplementary Table 2**. A total of 1467 metabolites were annotated in MLs subjected to three drying treatments. As shown in **Figure 1A**, the metabolites be classified into eight different categories, namely shikimates and phenylpropanoids (349, 23.79%), terpenoids (301, 20.52%), fatty acids (207, 14.11%), alkaloids (117, 7.98%), polyketides (64, 4.36%), amino acids and peptides (35, 2.39%), carbohydrates (21, 1.43%) and others (373, 25.43%).

### 3.2 Multivariate statistical analysis

Unsupervised PCA was performed to evaluate the overall metabolic differences among MLs processed by the three drying treatments and to assess the clustering and variability of biological replicates within each group (**Figure 1B**). The result of PCA was found that there was a significant separation between groups, indicating significant differences among the dried MLs. In addition, it was demonstrated that each group had good reproducibility due to tight clustering of its three biological replicates. Specifically, the first two principal components, PC1 and PC2, explained a cumulative variance of 76.01%, with PC1 and PC2 contributing 44.38% and 31.46%, respectively. Particularly, PC1 effectively separated FD from HD and SD, whereas PC2 clearly distinguished SD from FD and HD. In summary, PCA analysis revealed that the drying treatments significantly influenced the metabolic profiles of MLs.

### 3.3 Identification of differential metabolites

**Figure 2:**
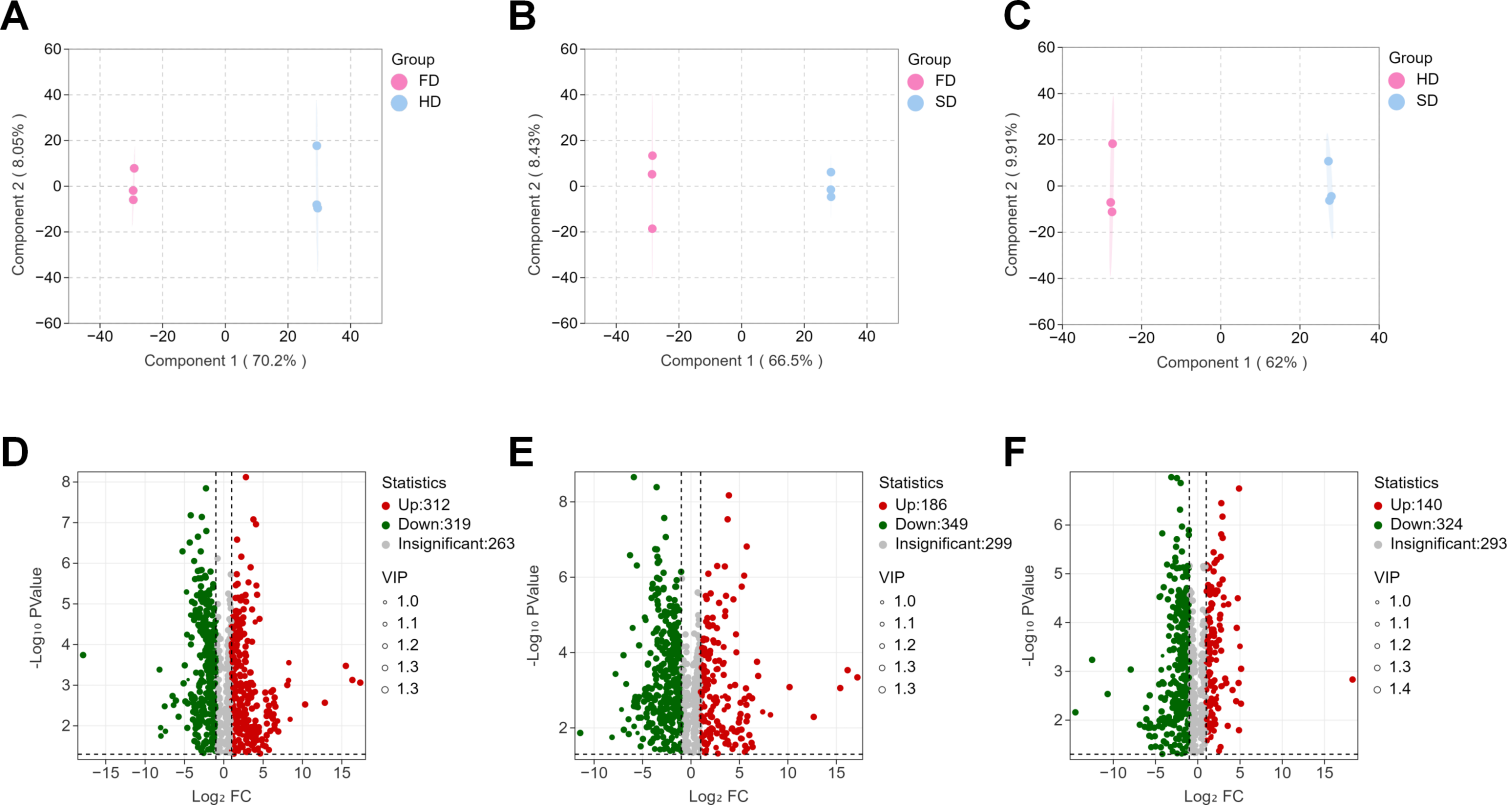
OPLS-DA score plots **(A–C)** and volcanic plots of differential metabolites (VIP ≥ 1 and fold change ≥ 2 or ≤ 0.5) **(D–F)**. **(A–C)** was OPLS-DA score plot of FD vs HD, FD vs SD, HD vs SD, respectively. **(D–F)** was volcanic plots of FD vs HD, FD vs SD, HD vs SD, respectively.

To further screened the differential metabolites of FD, HD and SD, the OPLS-DA was used. The permutation test was performed to assess the reliability of the model and exclude overfitting. It was found that the R2Y value reached 1.0 and Q2 values exceeded 0.9 in all pairwise comparisons, indicating that the OPLS-DA models were suitable for analysis (**Supplementary Figure 1**). OPLS-DA revealed differences among FD, HD and SD, thus the differential metabolites were identified based on criteria (VIP ≥ 1 and fold change ≥ 2 or ≤ 0.5) and visualized using volcano plots (**Figure 2**).

In FD vs HD, FD vs SD and HD vs SD groups, there were 631, 535 and 464 significantly differential metabolites, respectively (**Supplementary Table 3**). It was indicated that the treatment temperature influenced the metabolite composition of MLs largely. Notably, a significant proportion of the identified differential metabolites were classified as flavonoids, phenolic acids, alkaloids, and coumarins. These compounds are well-recognized for their anti-oxidant property and are considered the primary contributors to the health benefits of these plants.

**Figure 3:**
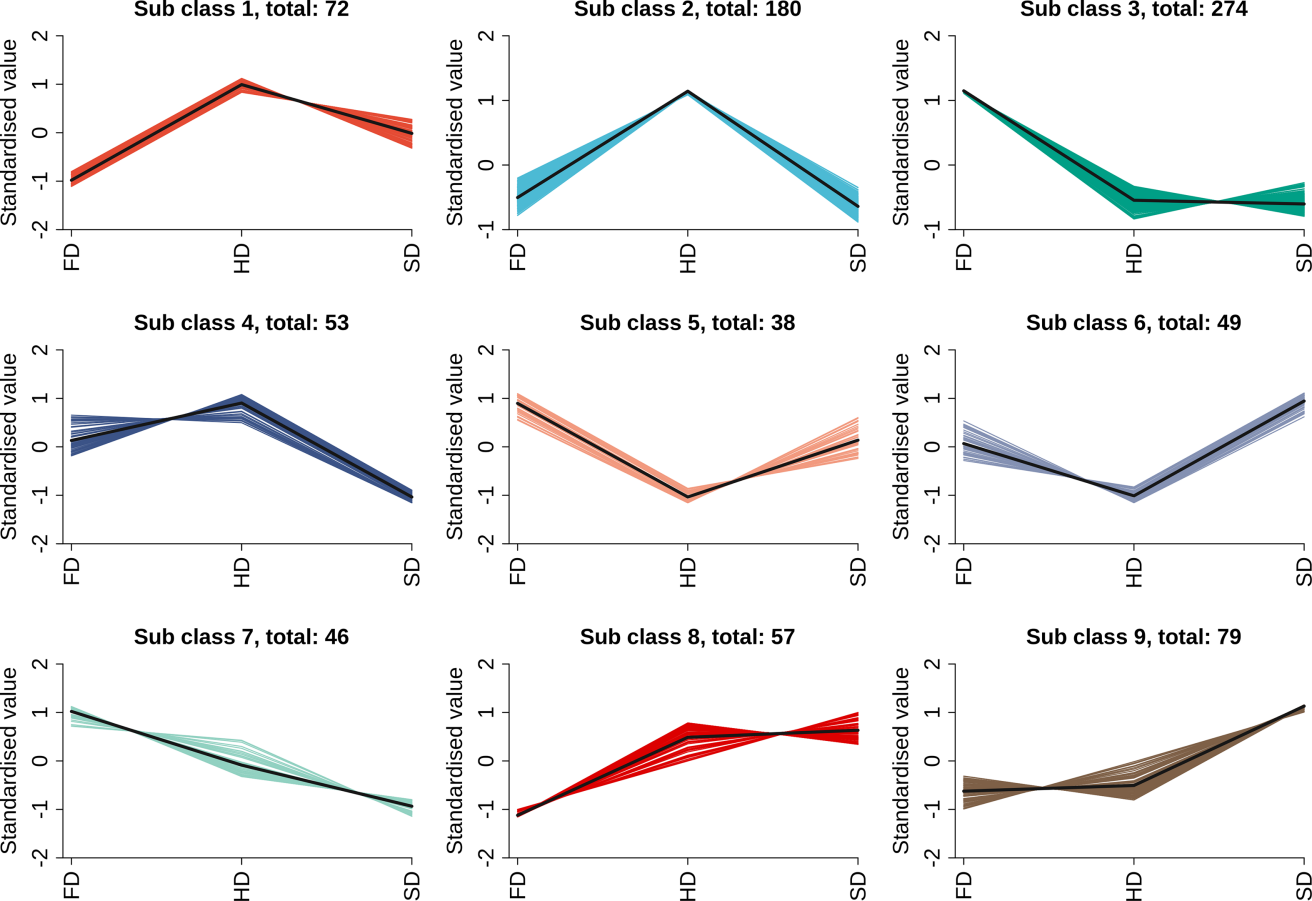
K-means clustering analysis.

To further explore the effect of temperature on responses of differential metabolites, the K-mean analysis was performed (**Figure 3**). Clusters 8 and 9 included 57 and 79 differential metabolites, respectively, whose levels increased progressively with processing temperature. In contrast, cluster 3 and 7 had 274 and 46 differential metabolites, respectively. The level of these metabolites declined as the temperature increased. Additionally, clusters 1, 2, and 4 comprised 305 differential metabolites that exhibited the highest levels in the HD group. Conversely, clusters 5 and 6 included 87 differential metabolites which displayed the lowest levels in the HD group. Thus, the combined total of 392 differential metabolites were collectively referred to as HD-specific metabolites.

Notably, a large number of phenolic compounds within the HD-specific metabolites were upregulated. For example, the contents of kaempferol and dihydrocurcumin in the HD were 1.59 and 3.69 times those of the FD, respectively.

### 3.4 Antioxidant capacity of dried MLs

**Figure 4:**
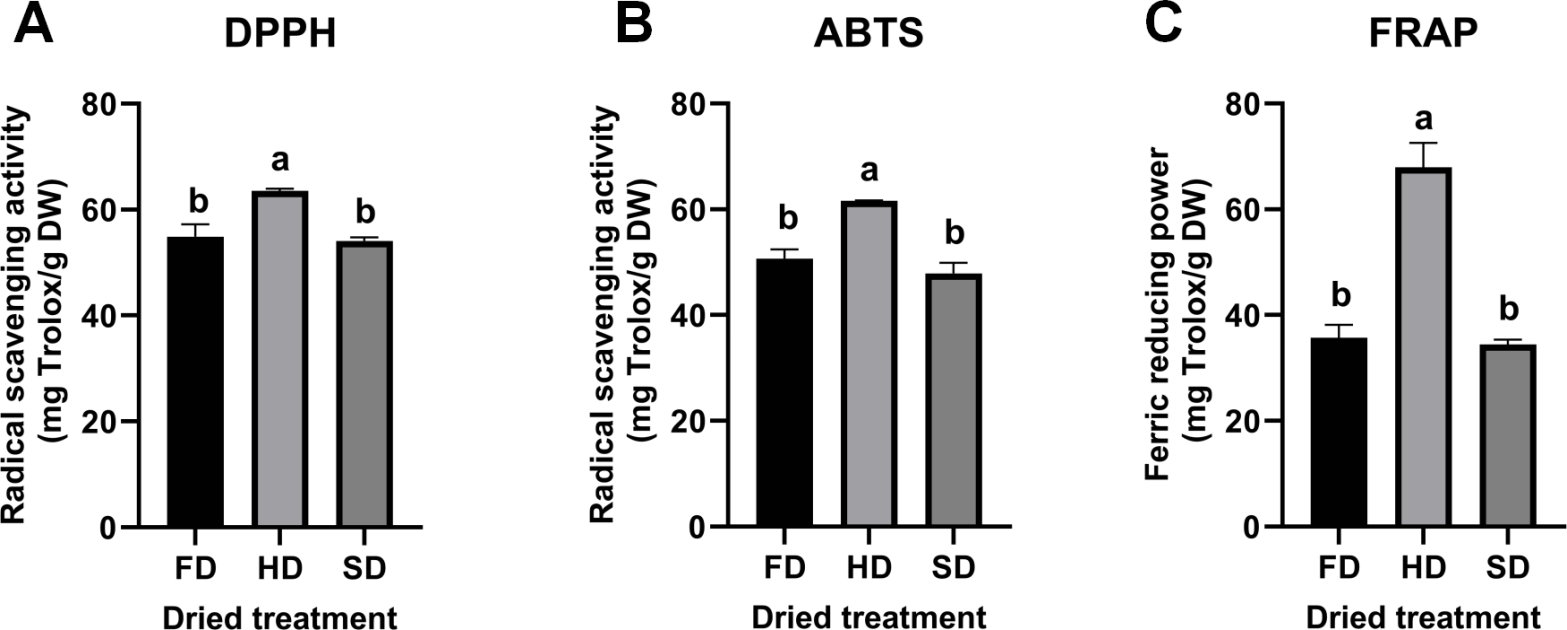
Antioxidant properties of MLs treated by different drying methods: **(A)** DPPH radical scavenging capacity, **(B)** ABTS radical scavenging capacity, and **(C)** Ferric reducing antioxidant power. Different lowercase letters indicated significant differences between groups at P < 0.05 according to Tukey's multiple comparison tests.

MLs, a food-medicine homologous substance, were widely consumed due to their excellent antioxidant and hypoglycemic properties. To comprehensively evaluate the effect of drying process on the antioxidant activity of MLs, DPPH and ABTS radical scavenging assays as well as the FRAP assay were performed. The same trend was observed across the three antioxidant capacity assays (**Figure 4**). HD consistently exhibited the strongest antioxidant activity, reaching 61.62 to 67.94 mg Trolox/g DW, which was significantly higher than those of FD and SD (*P* < 0.05). The antioxidant activity of FD (35.68~54.87 mg Trolox/g DW) was slightly higher than that of SD (34.41~54.08 mg Trolox/g DW), however, the difference between them was not statistically significant (*P* > 0.05). To identify metabolites related to antioxidant capacity, Pearson correlation analysis was utilized (**Supplementary Table 4**). These 212 identified metabolites showed strong positive correlations (r > 0.8, *P* < 0.01) with the *in vitro* antioxidant assays, suggesting they might contribute to the observed antioxidant effects. Specifically, these metabolites were classified into shikimates and phenylpropanoids (94, 44.34%), terpenoids (36, 16.98%), fatty acids (4, 1.89%), alkaloids (17, 8.02%), polyketides (11, 5.19%), amino acids and peptides (7, 3.30%), carbohydrates (1, 0.47%) and others (42, 19.81%). Many of these compounds had been proven to possess strong antioxidant activity, for instance, kaempferol (Sekiguchi et al., 2019), myricanol (Gashaw et al., 2024) and rutin (Rahmani et al., 2023).

**Figure 5:**
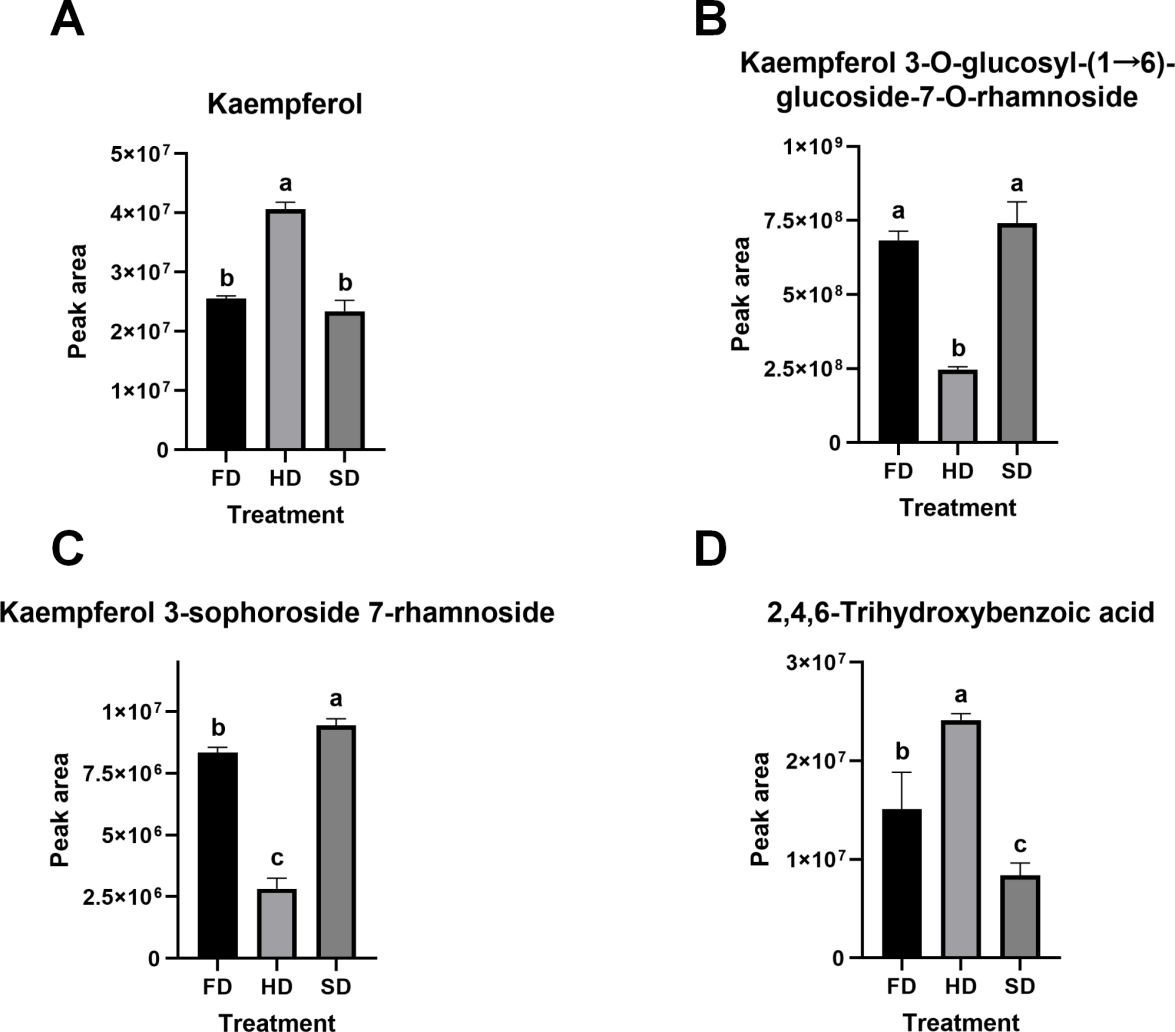
Quantitative results of seven antioxidant-related compounds in dried MLs. **(A)** Kaempferol; **(B)** Kaempferol 3-O-glucosyl-(1→6)-glucoside-7-O-rhamnoside; **(C)** Kaempferol 3-sophoroside 7-rhamnoside; **(D)** 2,4,6-Trihydroxybenzoic acid. Different lowercase letters indicated significant differences between groups at P < 0.05 according to Tukey's multiple comparison tests.

Consistent with these findings, this study on frost-bitten MLs showed that thermal processing at 60 °C induced the extensive degradation of complex glycosides, leading to the accumulation of their corresponding aglycones and simple glycosides. Specifically, kaempferol significantly accumulated in the HD group, whereas its glycosides, namely kaempferol 3-sophoroside 7-rhamnoside and kaempferol 3-glucoside- (1→6) -glucoside-7-rhamnoside, were significantly reduced (**Figures 5A–C**). Furthermore, there was an increased production of several flavonoids and their aglycones, including 2,4,6-trihydroxybenzoic acid (**Figure 5D**).

### 3.5 Network pharmacology analysis

#### 3.5.1 Target screening and construction of “compounds-targets-pathways” network

**Figure 6:**
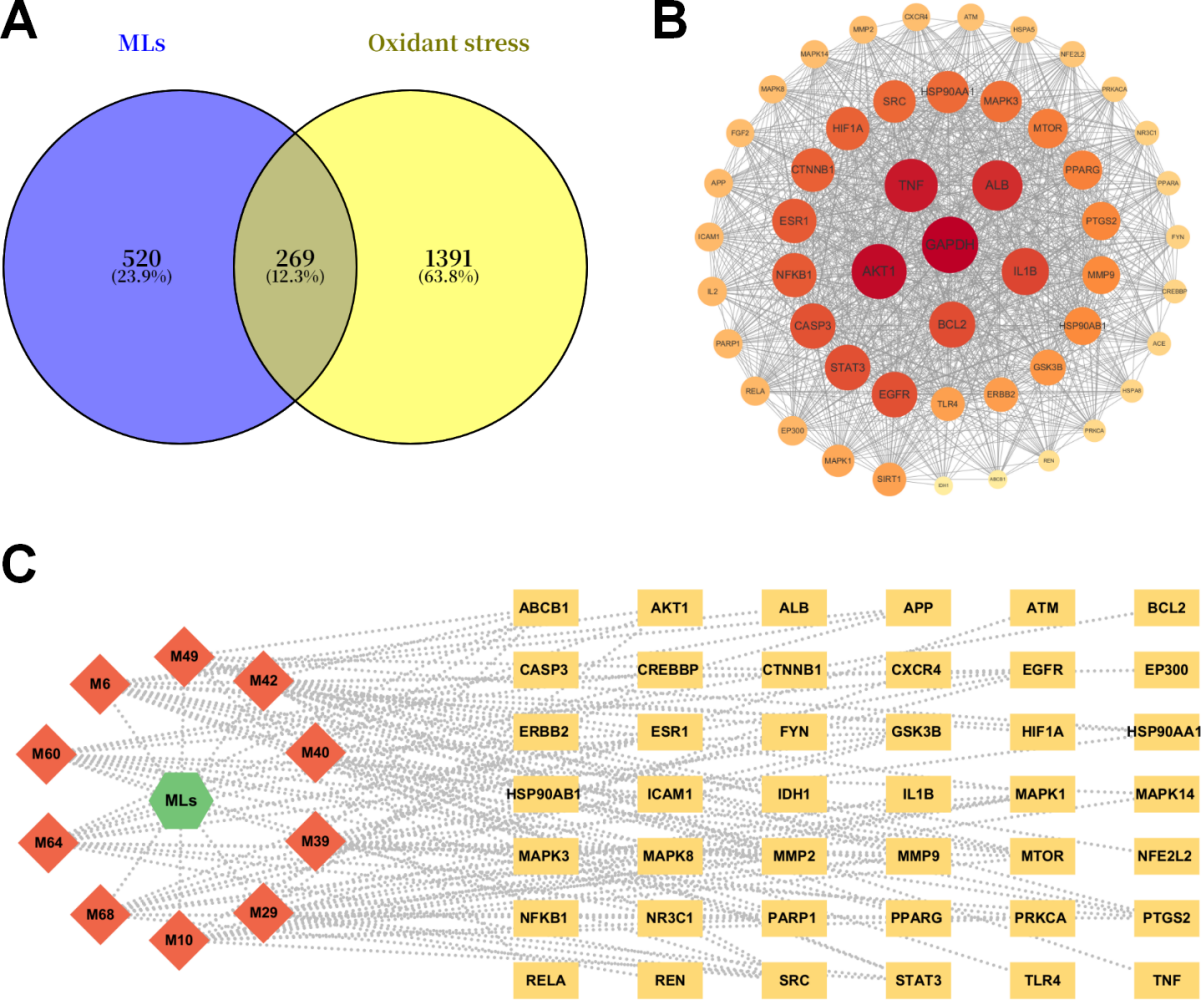
**(A)** Venn diagram of key metabolite targets versus oxidant stress related targets; **(B)** “protein-protein interactions” network diagram; and **(C)** “MLs-compounds-targets” network diagram.

A total of 89 compounds with potential antioxidant activity were screened from those showing positive correlations with all three antioxidant assays, including shikimates and phenylpropanoids (70), terpenoids (3), alkaloids (5), polyketides (8), carbohydrates (1) and others (2) (**Supplementary Table 5**). A total of 789 unique metabolite targets were obtained after integrating all targets with a “probability ≥ 0.1” and removing duplicates (**Supplementary Table 6**). Furthermore, genes from the GeneCards database were filtered using a median relevance score cutoff and supplemented with targets retrieved from OMIM and TTD, resulting in a final total of 1,660 targets associated with oxidative stress (**Supplementary Table 7**). Venn analysis identified 269 common targets shared between MLs and oxidative stress (**Figure 6A**). To further investigate the important role of MLs on the oxidative stress, the PPI analysis was performed. The Cytoscape 3.10.0 was used to evaluate the degree, closeness and betweenness centrality of the common targets, and targets with scores higher than the average were selected as core targets, resulting in 51 candidates. The **Figure 6B** showed that there were 51 nodes and 1055 edges. These core targets, associated with 58 compounds, mediate critical interrelated reactions that were highly relevant to oxidative stress. PPI network analysis revealed that the antioxidant effects of MLs were largely mediated through key targets, including GAPDH, AKT1, TNF, ALB, IL-1B, and BCL2.

It is worth noting that due to the limitation of sample size, the correlation analysis in this study was employed as a preliminary screening strategy. In order to further explore the interactions between critical antioxidant metabolites and targets, a “MLs-metabolites-targets” network was constructed. Based on the node connectivity values, the top ten metabolites were shown in **Figure 6C**. These metabolites were identified as 1,5-bis(4-hydroxy-3-methoxyphenyl)-1,4-pentadien-3-one (M6), myricanol (M10), guieranone A (M60), kaempferol (M64), lythramine (M68), cyclo(Pro-Tyr) (M29), 7,8-(2,2-dimethylpyrano)-3,4’-dihydroxy-5-methoxyflavan (M39), 7-hydroxy-5-methoxy-6,8-dimethylflavanone (M40), 7-prenyloxyaromadendrin (M42), cratoxyarborenone E (M49).

#### 3.5.2 GO enrichment analysis and KEGG signaling pathway analysis

**Figure 7:**
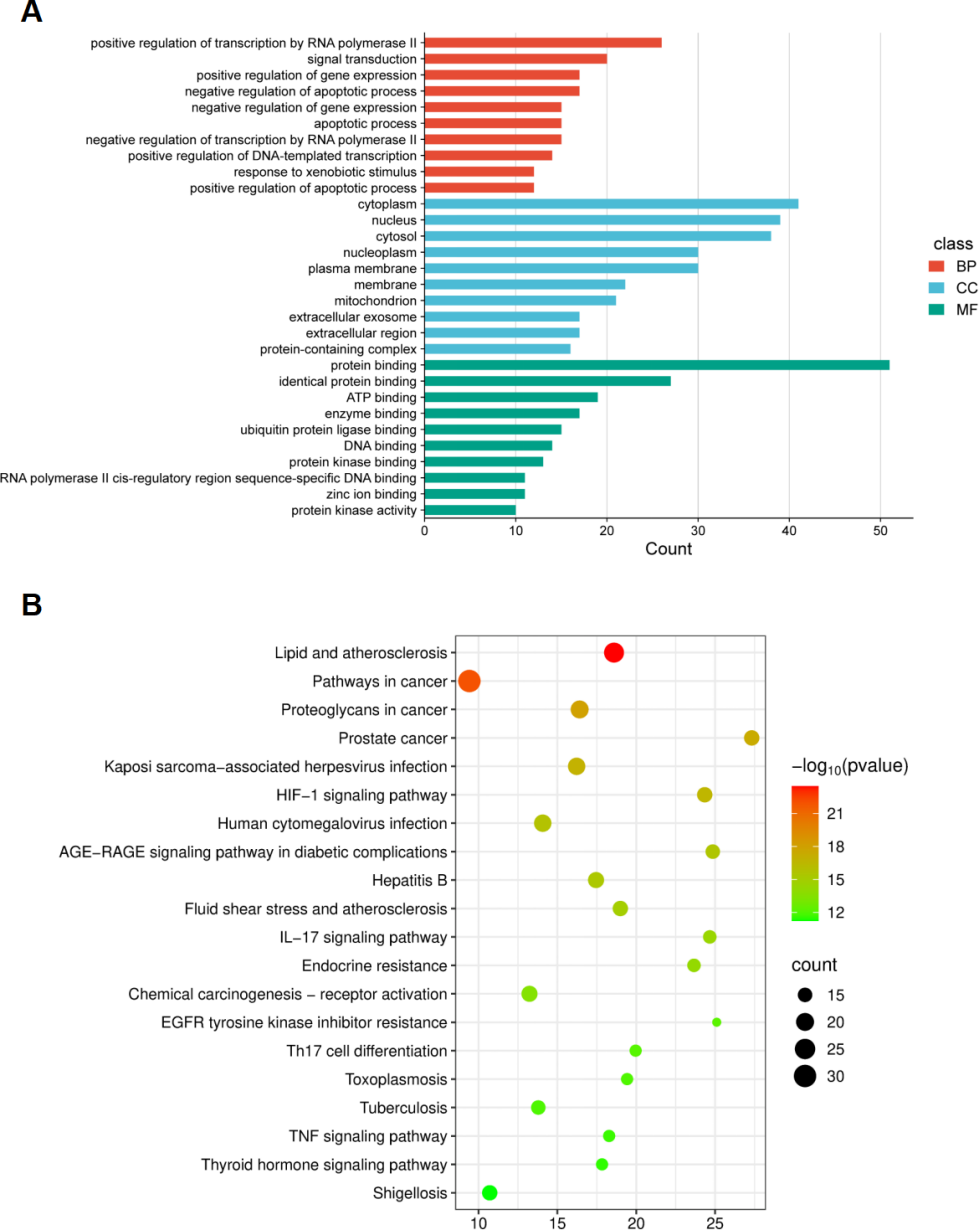
**(A)** GO functional enrichment analysis result (top 10) and **(B)** KEGG signaling pathway enrichment analysis result (top 20).

To further investigate the mechanisms underlying the protective effect against oxidative stress of MLs, GO functional and KEGG pathway enrichment analysis on the 51 core target genes was conducted using DAVID database. It was found that 495, 65, and 150 GO entries related to biological process (BP), cellular component (CC), and molecular function (MF), respectively. The top 10 significantly enriched entries are visualized in **Figure 7A**. Specifically, the BP analysis indicated that the targets were predominantly associated with regulation of transcription by RNA polymerase II, signal transduction, regulation of gene expression, apoptotic process, positive regulation of DNA-templated transcription, response to xenobiotic stimulus. In terms of CCs, notable enrichment was observed in cytoplasm, nucleus, cytosol, nucleoplasm, plasma membrane, membrane, mitochondrion. Regarding MFs, the enriched terms mainly involved protein binding, ATP binding, enzyme binding, ubiquitin protein ligase binding, DNA binding, RNA polymerase II cis-regulatory region sequence-specific DNA binding, zinc ion binding as well as protein kinase activity.

Moreover, this analysis revealed a total of 172 signaling pathways that were associated with oxidative stress. The top 20 KEGG pathways based on *P*-values were displayed in **Figure 7B**. Of particular interest, the targets related to oxidative stress were predominantly enriched in HIF-1 signaling pathway, AGE-RAGE signaling pathway in diabetic complications, TNF signaling pathway, chemical carcinogenesis-receptor activation, fluid shear stress and atherosclerosis.

#### 3.5.3 Molecular docking

**Figure 8:**
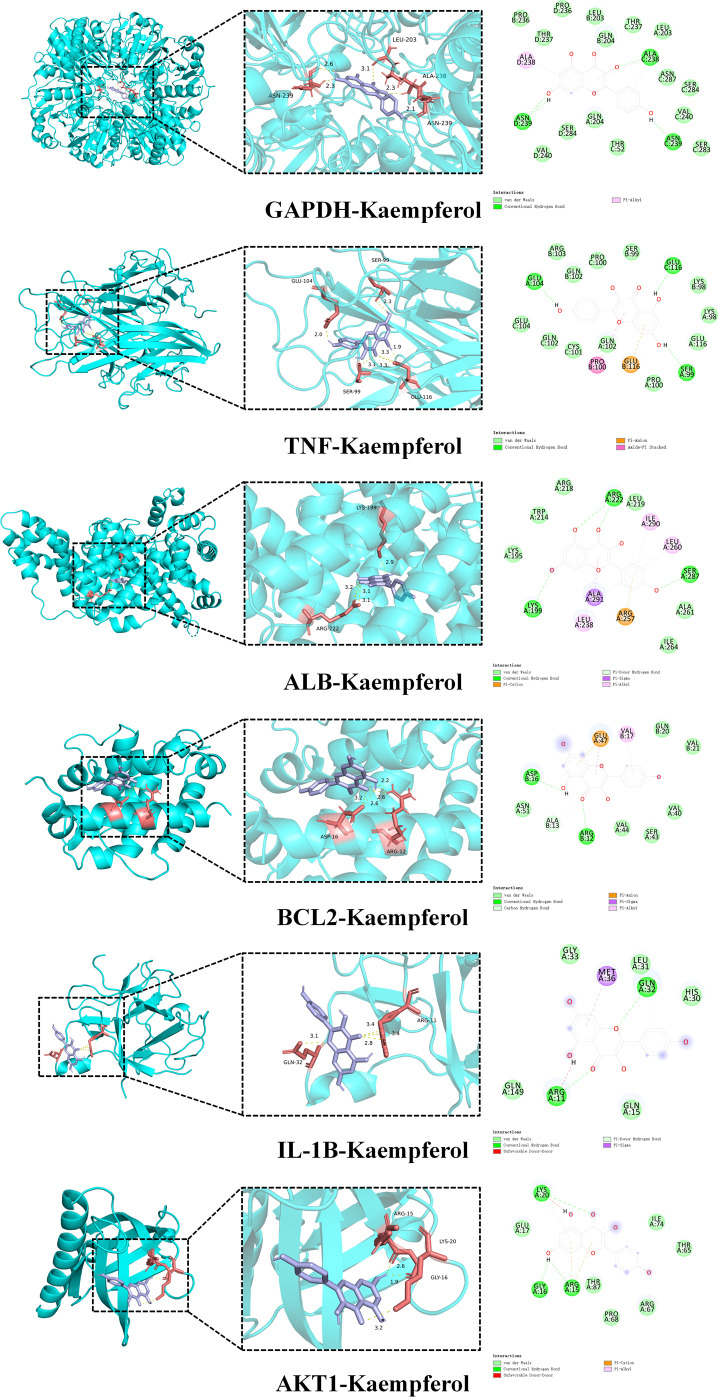
Molecular docking of kaemferol and targets.

1,5-bis(4-hydroxy-3-methoxyphenyl)-1,4-pentadien-3-one, myricanol, and kaempferol were selected as ligands for the molecular docking analysis. The core target proteins (GAPDH, AKT1, TNF, ALB, IL-1B, and BCL2) were chosen as receptors. Generally, when the docking energy is less than zero, the ligand and receptor protein can spontaneously bind, and a lower binding energy indicates a more stable interaction. As shown in the **Supplementary Table 8**, the binding energies of 1,5-bis(4-hydroxy-3-methoxyphenyl)-1,4-pentadien-3-one, myricanol, and kaempferol with all target proteins were lower than -6.0 Kcal/mol. In particular, kaempferol exhibited the lowest binding energies with GAPDH (-8.0 Kcal/mol), TNF (-6.4 Kcal/mol), ALB (-7.2 Kcal/mol) and BCL2 (-7.2 Kcal/mol). As illustrated in **Figure 8**, kaempferol stably binds to the distinct binding pockets of GAPDH, BCL2, TNF, IL-1B, ALB, and AKT1. Hydrogen bonds and hydrophobic interactions serve as the primary driving forces.

## 4 Discussion

MLs, a food-medicine homologous substance, were widely consumed due to their excellent antioxidant and hypoglycemic properties. As stated above, the HD had strongest antioxidant capacity. Although freeze-drying was generally considered the best treatment for preserving bioactive compounds, several studies had reported superior antioxidant activity in hot-air dried plant materials compared to their freeze-dried counterparts. For instance, higher antioxidant capacity was observed in hot-air dried pumpkin flour due to the formation of Maillard reaction products, or precursors of phenolic molecules by non-enzymatic interconversion between phenolic molecules (Que et al., 2008). Similarly, it was found that thermal treatment disrupted the plant cell wall and facilitated the release of bound phenolics in Asian pears, thereby enhancing their antioxidant activity (Jiang et al., 2019). In this study, it was found that hot-air drying promoted the hydrolysis of kaempferol glycosides, resulting in the production of free kaempferol. Compared to its glycosides, kaempferol displayed stronger antioxidant ability in ABTS and DPPH assays, as well as in other antioxidant evaluation systems (Guo et al., 2020). Typically, free forms of phenolic compounds exhibited greater antioxidant activity than their glycosylated derivatives due to reduced steric hindrance and increased exposure of active phenolic hydroxyl groups (Xiao et al., 2021). Furthermore, there was an increased production of 2,4,6-trihydroxybenzoic acid. This was attributed to the fact that the degradation of the aglycones involved the fission of the central C-ring, which released 2,4,6-trihydroxybenzaldehyde (and 2,4,6-trihydroxybenzoic acid) from the A-ring moiety (Seeram et al., 2001). These small molecule compounds also possessed strong antioxidant activity. In addition, hot-air drying led to a significant accumulation of LPC (18:4), which was commonly associated with membrane lipid remodeling and disruption of cellular membranes (Mishima et al., 2004). Such membrane perturbation might facilitate the leakage and diffusion of intracellular phenolic compounds. It was suggested that hot-air drying disrupts the cellular matrix, particularly membrane integrity, and might also indirectly affect cell wall–associated barriers, thereby promoting the release of phenolic compounds and improving their extraction efficiency, which consequently enhanced the overall antioxidant activity.

GAPDH, AKT1, TNF, ALB, IL-1B, and BCL2 were found to be related to the antioxidant activity of MLs through network pharmacology analysis. GAPDH was one of the most sensitive cellular targets for ROS and RNS. When reversible oxidative thiol modification was applied to GAPDH, glycolysis was inhibited, thereby allowing the metabolic flux to be transferred through the pentose phosphate cycle, and increasing the production of NADPH (Hildebrandt et al., 2015). For instance, treating boar sperm with rosmarinic acid to maintain their activity resulted in an increased activity of GAPDH (Feng et al., 2020). AKT1 was a serine/threonine protein kinase and plays a key role in regulation of the PI3K/Akt signaling pathway (Arciuch et al., 2009). In addition to modulating fundamental cellular activities such as proliferation, metastasis, and metabolism, AKT1 played a pivotal role in the regulation of inflammation and antioxidant stress (Yu et al., 2022; Lv et al., 2024). Consistently, a previous study also identified GAPDH and AKT1 as the primary antioxidant targets of *Fritillaria*, further substantiating the reliability of these two targets in alleviating oxidative stress (Guo et al., 2025). Tumor necrosis factor (TNF) and interleukin-1B (IL-1B) were intracellular pro-inflammatory factors. A decrease in their concentrations could directly affect the generation of reactive oxygen species (ROS) and reactive nitrogen species (RNS) in inflammatory cells, or indirectly regulate the activity of the signaling pathways stimulated by ROS (Piechota-Polanczyk and Fichna, 2014). Previous study found that flavonoids regulated gene expression by targeting transcription factors such as NF-κB, GATA-3, and STAT-6, thereby reducing the transcription of pro-inflammatory genes (Maleki et al., 2019). Similarly, in this study, STAT-6 was also identified as a core target in the intersection of MLs and oxidative stress. BCL-2 was widely recognized as a pivotal anti-apoptotic protein which regulates the intrinsic mitochondrial pathway of apoptosis. Impaired function of BCL-2 activated the mitochondrial-related cell death pathways (intrinsic apoptosis). On the other hand, BCL-2 maintained or augmented cellular antioxidant defense capacity that involved antioxidant enzymes (e.g. CAT, SOD, GPx, and GR) and an antioxidant molecule GSH (Jang and Surh, 2003). Albumin (ALB) played an important role in maintaining redox homeostasis. It scavenged ROS through its redox-active thiol groups. It was found that insect tea extract exerted a prophylactic effect against hepatic injury by enhancing antioxidant capacities. Concretely, the 200 mg/kg ITE increased levels of proteins such as ALB (Liao et al., 2016). Therefore, MLs might reduce inflammatory responses, eliminate excessive ROS, and decrease cell apoptosis by targeting GAPDH, AKT1, TNF, ALB, IL-1B, and BCL2, thereby further alleviating the oxidative stress in the body.

It was found that MLs regulated many pathways related to antioxidant and an-ti-inflammatory, such as HIF-1 signaling pathway and TNF signaling pathway. The HIF-1 signaling pathway play an important role in regulation of cell response to hypoxia that functions in inflammation, metabolism and apoptosis. HIF-1α, one of the subunits of the HIF-1 complex, can be activated by ROS through the inhibition of prolyl hydroxylases. Previous study found that cardamonin significantly inhibited the accumulation of ROS through the HIF-1α pathway, thereby ameliorating iron overload-induced oxidative stress in the body (Chen et al., 2024). Similarly, the HIF-1 signaling pathway was also identified to be involved in exerting the antioxidant capacity of citrus herb through network pharmacology (Cao et al., 2023). The TNF signaling pathway plays a pivotal role in regulating a wide spectrum of biological processes, including inflammation, immune response, cell proliferation, and apoptosis. It usually also involves the NF-κB signaling pathway. NF-κB activation induced by TNFα can directly affect ROS via increased expression of the antioxidant proteins, such as ferritin heavy chain (FHC) and manganese superoxide dismutase (MnSOD) (Morgan and Liu, 2010). It was indicated that the effect of MLs in attenuates oxidative stress was attributed to the coordinated regulation of multiple components, targets, and pathways, rather than being mediated by a solitary signaling mechanism.

The core compounds identified in this study possess strong physiological activity. 1,5-Bis(4-hydroxy-3-methoxyphenyl)-1,4-pentadien-3-one, a curcumin analogue, had been demonstrated in previous studies to possess potent antioxidant, anti-inflammatory, and antitumor activities (Suarez et al., 2010). Myricanol exhibits potent antioxidant activity, with an IC50 value of 13.48 μM in the DPPH radical scavenging assay, which is attributed to the enolic-OH moiety in its structure (Gashaw et al., 2024). In addition, another study had shown that myricanol improved neurodegeneration induced by oxidative stress. It was due to that the presence of two phenolic hydroxyl groups and one alcoholic hydroxyl group in the cyclic diarylheptanoid structure of myricanol contributed to its strong ROS scavenging ability and neuroprotective effects (Chen et al., 2017). Guieranone A is a compound with antibacterial activity (Silva and Gomes, 2003). Kaempferol is a type of flavonoid that exhibits powerful antioxidant, antimicrobial, anticancer, neuroprotective, and hepatoprotective activity (Bangar et al., 2023). Kaempferol effectively attenuated oxidative stress by downregulating HO-1 and NOX2 mRNA expression, while also inhibiting the release of key inflammatory and pro-fibrotic cytokines such as IL-6, TGF-β, and TNFα (Sekiguchi et al., 2019). In addition, kaempferol effectively mitigated lipid oxidation *in vivo*, thereby preventing the organs and cell structure from deterioration and protecting their functional integrity (Wu et al., 2018).

These findings demonstrated that the antioxidant active compounds in MLs could spontaneously bind to oxidative stress targets with favorable binding properties, which also validated the scientific reliability of the network pharmacology predictions. Combined with the network pharmacology results, this study concluded that antioxidant active compounds, including kaempferol, 1,5-Bis(4-hydroxy-3-methoxyphenyl)-1,4-pentadien-3-one, myricanol, etc., further regulated HIF-1 signaling pathway and TNF signaling pathway primarily by targeting key proteins such as GAPDH, AKT1, TNF, ALB, IL-1B and BCL2 to exert their antioxidant effects.

It should be noted that the current integrated metabolomics had inherent limitations in resolving certain structural isomers. Consequently, some annotated metabolites might represent unresolved mixtures or co-eluting isomers rather than pure single compounds. Future targeted analysis is required to confirm these precise structures. On the other hand, although the combination of metabolomics, network pharmacology and molecular docking techniques had provided rich insights into the antioxidant mechanism of MLs, this study still presented certain limitations. These predicted targets and pathways still need to be further validated through more wet−lab experiments, which were worth further investigation. For example, protein expression levels could be evaluated via Western blotting, or specific inhibitors could be employed in an oxidative stress-induced cell model to ultimately confirm these precise pathways.

## 5 Conclusion

This study investigated the effects of drying methods on the metabolites and antioxidant activity of MLs, and clarified the correlation between the changes in key substances and antioxidant activity. This study detected a total of 1467 metabolites, including terpenoids, shikimates and phenylpropanoids etc. The hot air-dried MLs exhibited the strongest DPPH, ABTS free radical scavenging activity and FRAP, which was attributed to the degradation of kaempferol glycosides and the accumulation of kaempferol. Moreover, network pharmacology and molecular docking further revealed that the core component in MLs, like 1,5-bis(4-hydroxy-3-methoxyphenyl)-1,4-pentadien-3-one, myricanol and kaempferol exerted the effect of alleviating oxidative stress by targeting proteins such as AKT1, TNF, ALB, IL-1B, and BCL2 and further influencing the HIF-1 and TNF pathways. This study provided guidance on the drying process of MLs, especially those that had been exposed to frost. However, future research should further explore the molecular mechanism through *in vivo* studies.

## Data Availability

The original contributions presented in the study are included in the article/**Supplementary Material**. Further inquiries can be directed to the corresponding authors.
