# Supplementary figures and images for: Integrated metabolomics, network pharmacology and molecular docking to reveal the effects of drying processes on the antioxidant activity of mulberry (Morus alba L.) leaves
Source: Front Plant Sci. 2026 Jun 8;17:1843809. doi: 10.3389/fpls.2026.1843809 (PMC13284004; doi:10.3389/fpls.2026.1843809)

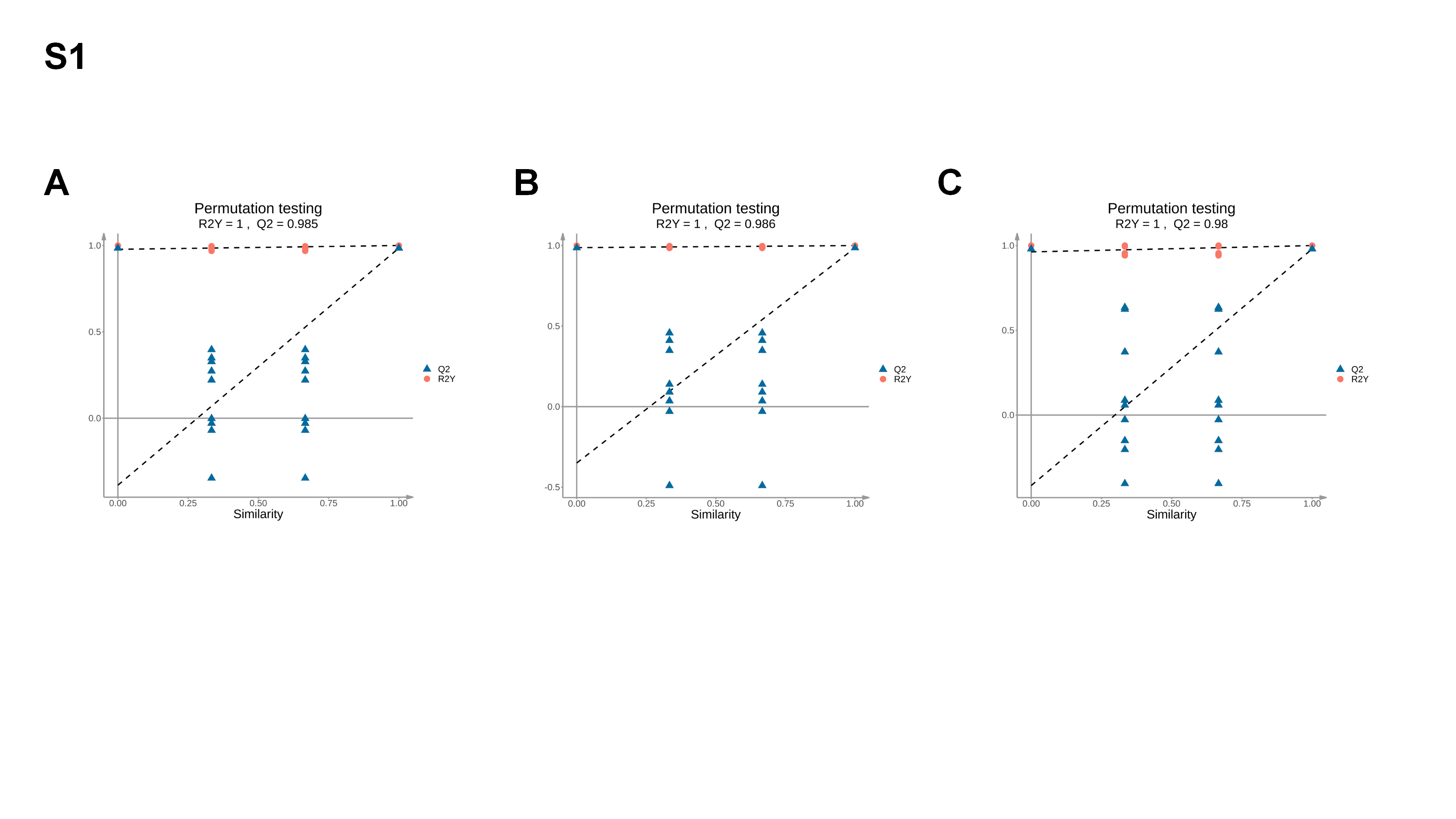

Supplement: Supplementary file 1 [file DataSheet1.zip › Supplementary Materials/Figure S1.tif]
